# Host Cell Oxidative Stress Promotes Intracellular Fluoroquinolone Persisters of Streptococcus pneumoniae

**DOI:** 10.1128/spectrum.04364-22

**Published:** 2022-11-29

**Authors:** Mirelys Hernandez-Morfa, Nicolás M. Reinoso-Vizcaíno, Nadia B. Olivero, Victoria E. Zappia, Paulo R. Cortes, Andrea Jaime, José Echenique

**Affiliations:** a Centro de Investigaciones en Bioquímica Clínica e Inmunología (CIBICI)-Consejo Nacional de Investigaciones Científicas y Técnicas (CONICET), Córdoba, Argentina; b Departamento de Bioquímica Clínica, Facultad de Ciencias Químicas, Universidad Nacional de Córdoba, Córdoba, Argentina; University of Pittsburgh

**Keywords:** *Streptococcus pneumoniae*, oxidative stress, stress response, intracellular survival, pneumocytes, macrophages, neutrophils, triggered-persistence, persistence, fluoroquinolones, moxifloxacin, levofloxacin, ciprofloxacin, host-pathogen interactions, pathogen interactions

## Abstract

Bacterial persisters represent a small subpopulation that tolerates high antibiotic concentrations without acquiring heritable resistance, and it may be generated by environmental factors. Here, we report the first antibiotic persistence mechanism in Streptococcus pneumoniae, which is induced by oxidative stress conditions and allows the pneumococcus to survive in the presence of fluoroquinolones. We demonstrated that fluoroquinolone persistence is prompted by both the impact of growth arrest and the oxidative stress response induced by H_2_O_2_ in bacterial cells. This process protected pneumococci against the deleterious effects of high ROS levels induced by fluoroquinolones. Importantly, S. pneumoniae develops persistence during infection, and is dependent on the oxidative stress status of the host cells, indicating that its transient intracellular life contributes to this mechanism. Furthermore, our findings suggest persistence may influence the outcome of antibiotic therapy and be part of a multistep mechanism in the evolution of fluoroquinolone resistance.

**IMPORTANCE** In S. pneumoniae, different mechanisms that counteract antibiotic effects have been described, such as vancomycin tolerance, heteroresistance to penicillin and fluoroquinolone resistance, which critically affect the therapeutic efficacy. Antibiotic persistence is a type of antibiotic tolerance that allows a bacterial subpopulation to survive lethal antimicrobial concentrations. In this work, we used a host-cell infection model to reveal fluoroquinolone persistence in S. pneumoniae. This mechanism is induced by oxidative stress that the pneumococcus must overcome to survive in host cells. Many fluoroquinolones, such as levofloxacin and moxifloxacin, have a broad spectrum of activity against bacterial pathogens of community-acquired pneumonia, and they are used to treat pneumococcal diseases. However, the emergence of fluoroquinolone-resistant strains complicates antibiotic treatment of invasive infections. Consequently, antibiotic persistence in S. pneumoniae is clinically relevant due to prolonged exposure to fluoroquinolones likely favors the acquisition of mutations that generate antibiotic resistance in persisters. In addition, this work contributes to the knowledge of antibiotic persistence mechanisms in bacteria.

## INTRODUCTION

Streptococcus pneumoniae naturally resides in the nasopharynx of humans and is a known agent of common infections, such as sinusitis and otitis, as well as severe disease states, including meningitis and community-acquired pneumonia (1). Despite the availability of antibiotics and vaccines, pneumococcal disease is responsible for the most deaths among vaccine-preventable diseases globally, causing more than 1 million deaths every year (2, 3). The global emergence of pneumococcal strains resistant to antibiotics (4) has made it increasingly difficult to treat invasive diseases caused by S. pneumoniae, particularly meningitis (5). FQs are an important treatment option for invasive pneumococcal disease, but it is threatened by the emergence of FQ-resistant strains (6). In S. pneumoniae, FQ resistance rises mainly via the stepwise accumulation of chromosomal mutations in the quinolone resistance-determining regions (QRDRs) of the *parC* (encodes topoisomerase IV) and/or *gyrA* (encodes DNA gyrase) genes (6).

In addition to antibiotic resistance, other adaptive mechanisms to tolerate antibiotics have been identified in bacteria. For instance, antibiotic tolerance is a phenomenon that allows a bacterial population to survive exposure to a bactericidal antibiotic concentration without an increase in the MIC. When this phenotype occurs only in a subpopulation, it corresponds to persistence. Heteroresistance is similar to persistence; however, heterotolerants can replicate in the presence of the antibiotic and present a higher MIC than the rest of the bacterial population (7). In S. pneumoniae, vancomycin tolerance was reported in clinical strains (8), which critically affects the therapeutic efficacy of clinical infections, as well as heteroresistance to penicillin (9) and fosfomycin (10).

Related to stress conditions, reactive oxygen species (ROS) can act as host defense antimicrobial factors that S. pneumoniae must overcome to infect host cells. During infection, S. pneumoniae produces H_2_O_2_ (11, 12) to compete with the respiratory tract microbiota, cause cellular damage (13), and suppress inflammasome-dependent innate immunity (14). It has been described that S. pneumoniae can survive in host cells that produce different levels of ROS, such as pneumocytes (15–17), macrophages (15, 18, 19), and neutrophils (20, 21). In parallel, this pathogen also triggers an oxidative stress response to tolerate the ROS produced endogenously or secreted by human cells (22, 23). S. pneumoniae is one of the most important bacterial producers of H_2_O_2_. More than 80% of endogenous H_2_O_2_ is synthesized by SpxB, a pyruvate oxidase, and the rest by LctO, a lactate oxidase. To tolerate high levels of H_2_O_2_, S. pneumoniae has no catalase, one of the most common ROS detoxifying enzymes. However, this pathogen has developed efficient mechanisms to protect itself, such as SodA (superoxide dismutase), TpxD (thiol peroxidase), and NOX (NADH oxidase), among others, as well as enzymes to repair oxidized proteins, such as HtrA (chaperone) and ClpP (protease) (22, 24).

FQs are one of the most used antibiotics to treat pneumococcal infections (25) that are related to the oxidative metabolism of S. pneumoniae. These antibiotics interact with topoisomerases (26), causing DNA damage and chromosome fragmentation (27). During FQ treatment, pneumococcal cells also increase iron transport, triggering the generation of ROS via the Fenton reaction and contributing to the lethality of FQs, as reported in other bacteria (28), being ROS the main factor directing the postantibiotic effect that causes bacterial death (27, 29).

In this work, we report for the first time an antibiotic persistence mechanism in S. pneumoniae, which is prompted by previous exposure to oxidative stress conditions in bacterial cultures. We also demonstrate that the increased ROS levels in pneumococci-infected human cells, such as pneumocytes and phagocytes, are able to induce intracellular FQ persisters of S. pneumoniae. We propose that this ROS-triggered persistence is clinically relevant since extended exposure of FQ on bacterial cells may influence the generation of FQ-resistant mutants, as described in Escherichia coli (30).

## RESULTS

### Oxidative stress induces fluoroquinolone persistence in S. pneumoniae.

**FIG 1:**
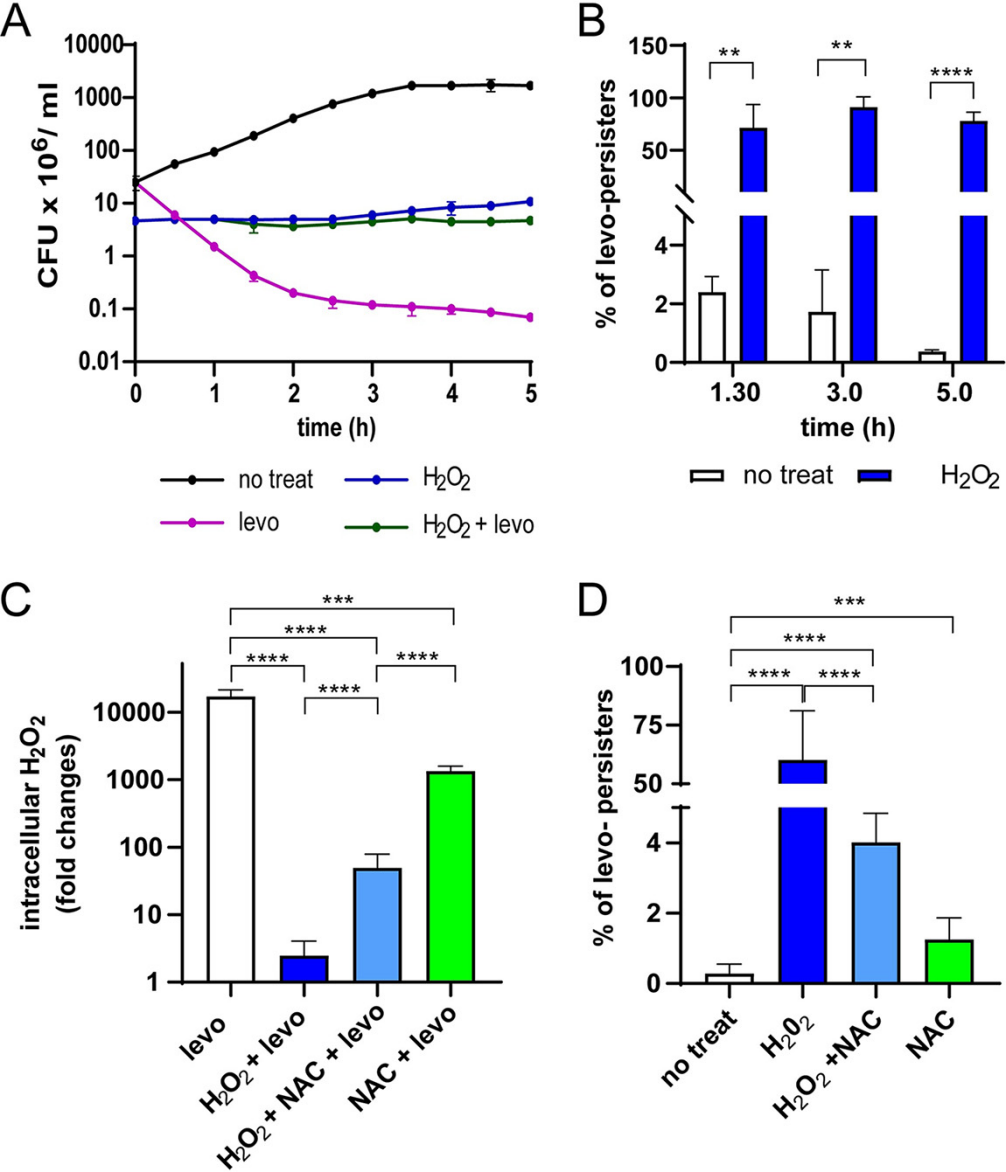
Pneumococcal FQ persistence is induced by oxidative stress in culture media. (A) The subpopulation that survives H_2_O_2_ tolerates levofloxacin. Growth curves represent CFU/mL values measured at different time points of bacterial cultures. Bacterial cells were grown to midlog phase, treated with either 20 mM H_2_O_2_ for 30 min, or 6 μg/mL levofloxacin for 5 h, or a combination of both treatments, and viable cells were counted. (B) The percentage of levofloxacin persisters induced by H_2_O_2_ at different time points. Percentages were calculated from the data shown in panel a, and represent the mean ± SEM of at least three replicates. (C) Bacterial cells were grown in BHI to midlog phase, exposed to either 10 mM NAC for 1 h, 20 mM H_2_O_2_ for 30 min, or NAC 10 mM for 1 h followed by 20 mM H_2_O_2_ for 30 min, and viable cells were counted. The intracellular H_2_O_2_ concentration of bacterial cells was measured by the classic horseradish peroxidase method, and values were expressed as μM of H_2_O_2_ found in 1× 10^6^ CFU. The fold variation was calculated as the ratio of intracellular H_2_O_2_ concentration in stressed cells versus untreated cells. (D) H_2_O_2_-induced levofloxacin persistence is affected by pretreatment with antioxidants. The NAC-treated cells showed inhibition of persistence to levofloxacin. The percentage of levo-persisters was determined by counting the CFU/mL values shown in Fig. S5 obtained after exposure to H_2_O_2_, NAC or NAC/H_2_O_2,_ with and without levofloxacin treatment for 5 h, and referred to those obtained at time zero. In both panels, values represent the mean ± SEM of at least three replicates. Statistical significance was determined using the two-tailed test and indicated as *P < *0.001 (**), *P < *0.001 (***) and *P < *0.0001 (****).

To determine the putative effect of an oxidative environment on the FQ treatment, bacterial cells from strain R801 were first exposed to 20 mM H_2_O_2_ for 30 min and then to either 6 μg/mL levofloxacin, 5 μg/mL moxifloxacin, or 2.5 μg/mL ciprofloxacin for 5 h. These concentrations correspond to the FQ levels found in human serum after antibiotic administration (31–33). We found that approximately 1% of the total cells were able to survive to H_2_O_2_ (Fig. 1A), and the levofloxacin-treated cells showed the typical killing curve, as reported (34) (Fig. 1A). Curiously, more than 85% of the H_2_O_2_-treated population was able to survive to 6 μg/mL levofloxacin (Fig. 1B). We also confirmed the persistence to 5.0 μg/mL moxifloxacin (Fig. S1A and B) and 2.5 μg/mL ciprofloxacin (Fig. S1C and D) in H_2_O_2_-treated cells. As mentioned, FQ persistence was detected in the R801 (derivate of R6), but we also detected the formation of levo-persisters in other pneumococcal strains, such as R6 (derivate of D39; Fig. S1E and F), D39 (serotype 2; Fig. S1G and H), Cp1015 (derivate of Rx1, serotype 3; Fig. S1I and J) (35), and TIGR4 (serotype 4; Fig. S1K and L). We also evaluated the effect of a non-FQ antibiotic, 10 μg/mL rifampicin (36) after H_2_O_2_ exposure, and a low persistence level to this antibiotic was detected, approximately 4–10 times less than FQs (Fig. S1M and N).

To corroborate that FQ persistence is a transient phenotype, the R801 strain was exposed to H_2_O_2_ and then to different FQs, as described above. Posteriorly, aliquots from each one of these cultures were recultured in BHI to the midlog phase and treated with the same FQ that was used in the initial culture. We found that S. pneumoniae was not able to recover persistence after regrowths (Fig. S2), and the MICs for each antibiotic were identical to the nontreated cells, confirming that this phenotype corresponds to FQ persistence.

Altogether, these results indicate that this phenomenon is independent of the pneumococcal strain and the FQ type used. Considering that this particular phenotype affects only a determined subpopulation that survives to H_2_O_2_, we propose it should be considered a triggered FQ persistence, as defined by Balaban et al. (7).

It is known that bacterial persistence is induced by many environmental factors (7). To assess the effect of another environmental stress, such as acidic pH, bacterial cells were exposed to acidified medium (MD at pH 5.2) for 2 h, and then to 6 μg/mL levofloxacin for 5 h. Under acidic conditions, we observed an increase of 37 times in FQ persistence; however, this phenotype was markedly lower than H_2_O_2_-treated cells, exhibiting a rise of 1,000 times compared with the non-H_2_O_2_-treated group (Fig. S3A, B and C).

To test whether inhibition of protein synthesis affects the induction of FQ persistence, we used 2.0 μg/mL chloramphenicol, a common protein-synthesis inhibitor. After 1 h of treatment with this antibiotic, cells were exposed to 6 μg/mL levofloxacin for 5 h. We found that the FQ persistence was inhibited in either chloramphenicol-treated or chloramphenicol/H_2_O_2_-treated cells, displaying decreases of 53 and 100 times, respectively (Fig. S3D, E and F), indicating that protein synthesis is required for this mechanism.

### Inhibition of bacterial ROS production impairs FQ persistence.

To corroborate that oxidative stress is an FQ persistence inducer, bacterial cells were treated with either H_2_O_2_ or N-acetylcysteine (NAC), a known antioxidant agent (37). We used a treatment of 10 mM NAC for 3 h that does not affect pneumococcal viability (17). NAC inhibited 72 times the endogenous H_2_O_2_ production in S. pneumoniae (Fig. S4A). We detected an increase of four times the intracellular ROS level in H_2_O_2_-treated cells, while cells first exposed to NAC and then to H_2_O_2_ displayed a decrease in the ROS level of 41 times compared with H_2_O_2_-treated cells (Fig. S4A).

Regarding the FQ effect on bacterial oxidative status, we confirmed that antibiotic exposure increased the endogenous H_2_O_2_ level more than 10,000 times, as previously reported (29). In contrast, a previous NAC exposure decreases 10 times this oxidative status in FQ-treated cells. Interestingly, we found that a previous H_2_O_2_ exposure inhibits 6,000 times the H_2_O_2_ production observed in FQ-exposed cells (Fig. 1C). The higher FQ persistence induced in H_2_O_2_-treated cells, the lower their endogenous H_2_O_2_ level. It was also observed that this phenotype was inhibited by cotreatment with NAC/H_2_O_2_ previous to FQ exposure (Fig. 1B and Fig. S4). These findings supported the idea that FQ persistence is caused by a marked drop in intracellular ROS level in S. pneumoniae that justifies the bacterial survival to FQ exposure.

### Fluoroquinolone persistence occurs in H_2_O_2_-induced growth-arrested pneumococci.

**FIG 2:**
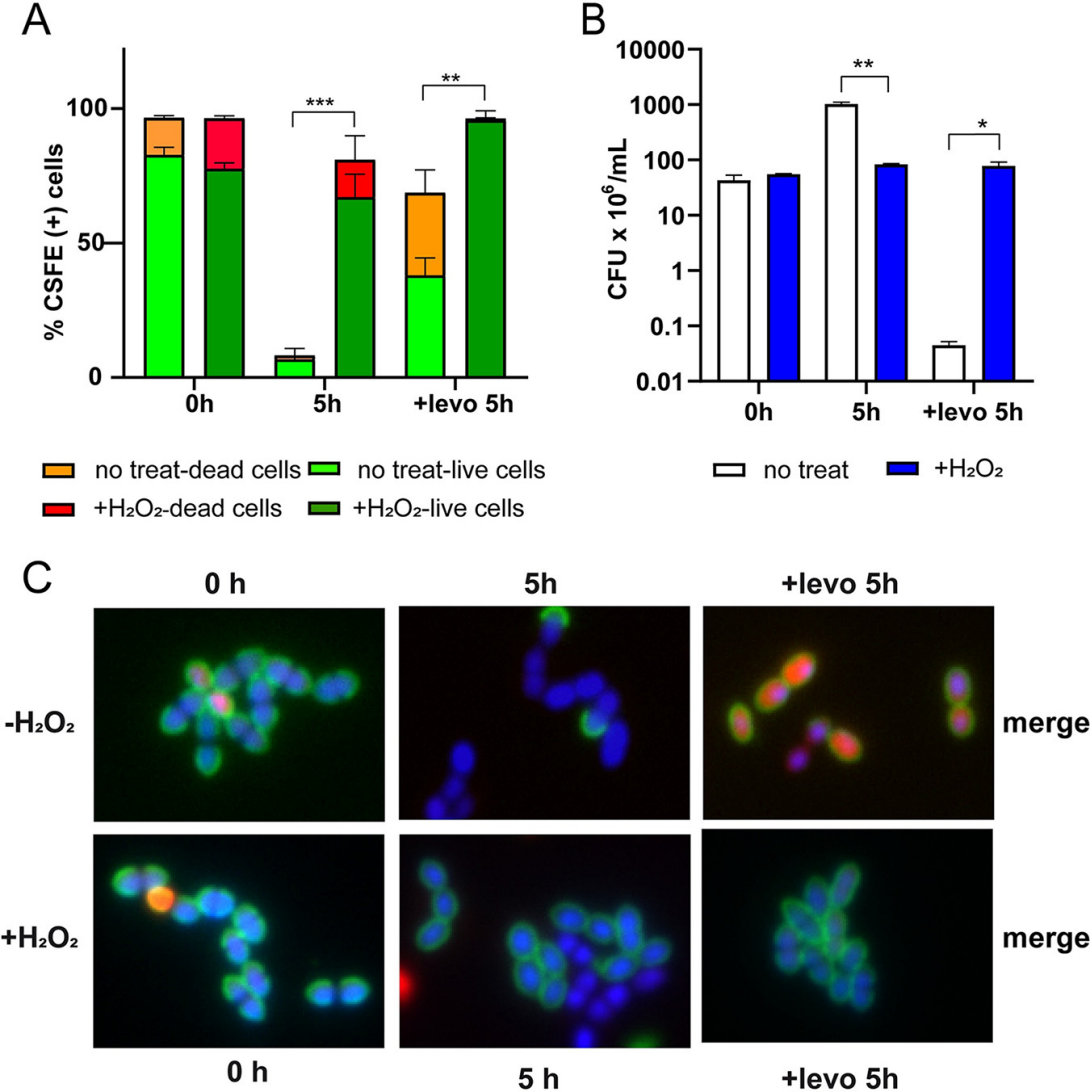
H_2_O_2_ treatment arrests growth in S. pneumoniae. Bacterial cells were grown in BHI to the midlog phase and exposed to 20 mM H_2_O_2_ for 30 min. After this treatment (referred to as time zero), cells were exposed to CSFE to label live pneumococci at the starting point (green), to Hoechst to label chromosomal DNA (blue) and to propidium iodide to label dead cells (red). Posteriorly, bacterial cells were incubated in BHI with 6 μg/mL levofloxacin for 5 h. Replicative cells lose the green signal due to CSFE labeling, which is not conserved after bacterial division. At each time point, cells were analyzed by high-content microscopy to simultaneously detect the three fluorescent signals. (A) H_2_O_2_-treated cultures show a higher percentage of CSFE-labeled cells. The percentages of CSFE- and PI-labeled cells were estimated at the total number of Hoechst-labeled cells. Live cells are indicated in light (nontreated) and dark (H_2_O_2_-treated) green. Dead cells are indicated in orange (nontreated) and red (H_2_O_2_-treated). (B) After levofloxacin treatment, H_2_O_2_-treated cells had higher CFU values than non-H_2_O_2_-treated cells. Data shown in both panels represent the mean ± SEM of at least three replicates. Statistically significant differences were determined using the two-tailed test and are indicated as *P < *0.05 (*), *P < *0.01 (**), *P < *0.001 (***) or *P < *0.0001 (****). (C) Representative images of bacterial cells stained with CSFE (green), Hoechst (blue), and propidium iodide (red) with samples taken from the assay described above.

To analyze the duplication capacity of S. pneumoniae, growth curves were obtained from cultures grown under stress conditions. We observed that the doubling times in pneumococci exposed to either 20 mM H_2_O_2_ or the double treatment with 20 mM H_2_O_2_ and 2.0 μg/mL chloramphenicol for 5 h were 3.8 and 2 times, respectively, higher than nontreated bacterial cells, while pneumococci exposed to pH 5.2 showed no changes (Fig. S5A and B). To prove the pneumococcal growth inhibition, we evaluated single slow-growing and growth-arrested cells. Bacterial cells were cultured in BHI containing either 20 mM H_2_O_2_, 6 μg/mL levofloxacin, or pretreated with H_2_O_2_ and then exposed to levofloxacin. Then, cells were washed and labeled with CFDA-SE, a nonfluorescent compound that is hydrolyzed by intracellular esterases to CSFE, a fluorescent tracer that covalently tags intracellular proteins, resulting in long-term cell labeling. In this assay, CFDA-SE was used to detect nonproliferating cells as this green fluorescent signal decreases as bacterial cells divide (38). In addition, cells were also labeled with propidium iodide (PI), a red-fluorescent cell viability dye that only permeates membranes of dead or damaged cells. After 5 h of incubation, all bacterial DNA was stained with Hoechst and used as a reference in the analysis performed by high-content microscopy. In the merged images, CSFE-labeled pneumococci without a positive signal of PI (CSFE[+]/PI[–]), are considered either slow-growing or growth-arrested cells (Fig. 2A). At time zero, the cell viability was a high percentage of CSFE(+)/PI(–). After 5 h of growth, nontreated pneumococci displayed a low percentage of CSFE(+)/PI(–) cells due to division events diluting the CSFE signal. In contrast, H_2_O_2_-treated pneumococci showed an increase of 7 times in growth-arrested cells compared to untreated cells. After levofloxacin treatment, 60% of the non-H_2_O_2_-exposed population showed CSFE(+)/PI(-) signals, which was coincident with higher CFU values compared with non-H2O2-treated cells (Fig. 2B) while the H_2_O_2_-exposed cells displayed near 95% of CSFE(+)/PI(–) (Fig. 2A). Representative images of the differential labeling are shown in Fig. 2C. Altogether, these results suggest that FQ persistence is induced by growth arrest, and it is induced by oxidative stress.

### Oxidative stress genes are involved in the FQ persistence mechanism of S. pneumoniae.

**FIG 3:**
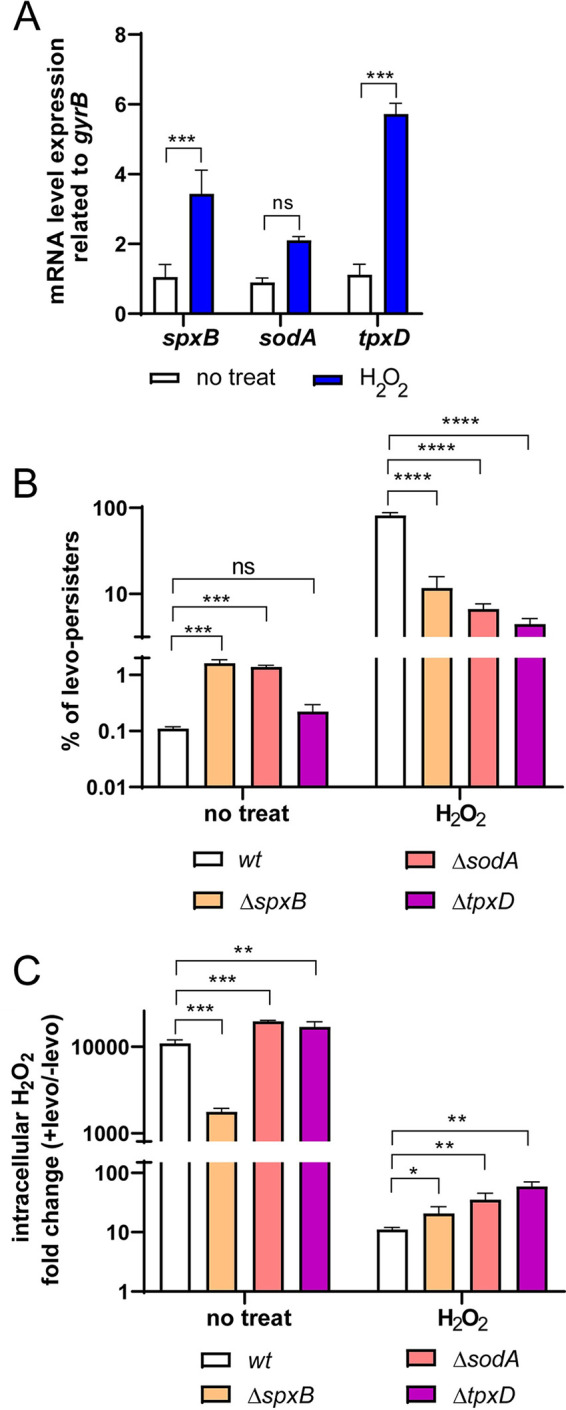
Oxidative stress genes are involved in the induction of FQ persistence. (A) H_2_O_2_-treated cells exhibit increased expression of the *spxB*, *sodA*, and *tpxD* genes. The R801 *wt* strain was grown in BHI to the midlog phase and exposed to 20 mM H_2_O_2_ for 30 min. Total RNA was purified and the transcript level of the *spxB*, *sodA* and *tpxD* genes was determined by qPCR. The fold change in gene expression was determined using transcript levels obtained in H_2_O_2_-treated and nontreated cells, and using the 2–ΔΔCT method. The *gyrB* gene was used as an internal control. The error bars represent mRNA level expression for three different experiments. (B) Mutations in oxidative stress genes block the induction of FQ persistence. The *ΔspxB*, *ΔsodA*, *ΔtpxD* and *wt* strains were grown in BHI until the midlog phase and exposed to 20 mM H_2_O_2_ for 30 min. The percentage of levo-persisters was determined by counting the CFU/mL values shown in Fig. S8 obtained after exposure to 6 μg/mL levofloxacin for 5 h, with and without previous exposure to 20 mM H_2_O_2_. (C) When exposed to exogenous H_2_O_2_, the Δ*spxB*, Δ*sodA*, and Δ*tpxD* mutants have higher intracellular H_2_O_2_ concentrations than the *wt* strain. The intracellular H_2_O_2_ concentration was measured as described in Fig. 1C legend. The fold variation was calculated as the difference concerning H_2_O_2_ concentration determined in levo-treated versus nontreated cells. In all panels, values represent the mean ± SEM of at least three replicates. Statistical significance was determined using the two-tailed test and indicated as *P < *0.05 (*), *P < *0.01 (**), *P < *0.001 (***) or *P < *0.0001 (****).

Because FQ persistence depends on previous exposure to H_2_O_2_, we hypothesized that the oxidative stress response of S. pneumoniae (22) is involved in the induction of this mechanism. By qPCR assays, we assessed the expression of different oxidative stress genes, such as *spxB* (encodes pyruvate oxidase that produces more than 80% of the total H_2_O_2_) (11), *sodA* (encodes a superoxide dismutase that degrades superoxides) (22), and *tpxD* (encodes a peroxiredoxin that degrades H_2_O_2_) (39) in response to 20 mM H_2_O_2_. In H_2_O_2_-treated cells, we observed that the expression of *spxB* and *sodA* increased 2 times, while the *tpxD* transcripts showed a 7-fold increment (Fig. 3A). To confirm that these genes are involved in the oxidative stress response, we generated individual mutants and evaluated bacterial survival after 20 mM H_2_O_2_ treatment. All three mutants were more susceptible to H_2_O_2_ than *wt*, as described (11, 39–41) (Fig. S6).

To determine the putative role of these genes, we evaluated FQ persistence in each mutant obtained. With a previous H_2_O_2_ treatment and further FQ exposure, all three mutants showed lower FQ persistence levels than *wt* (Fig. 3B and Fig. S6). In media containing 6 μg/mL levofloxacin, the Δ*spxB* mutant showed a decrease of 100 times in the endogenous H_2_O_2_ production, as described (11), while the Δ*sodA* and Δ*tpxD* mutants slightly increased their H_2_O_2_ level relating to *wt* due to a lack in H_2_O_2_ detoxification activity (Fig. 3C). This phenotype is inversely correlated with the persister generation, as shown by comparison between the H_2_O_2_-treated cells (Fig. 3B versus Fig. 3C), the more endogenous H_2_O_2_ was produced by FQ exposure, the fewer FQ persisters generated in H_2_O_2_-treated mutant pneumococci. Altogether, these results indicated that the FQ persistence mechanism depends on the intracellular ROS levels controlled by an active oxidative stress response.

### FQ persistence is induced by host cell oxidative stress.

**FIG 4:**
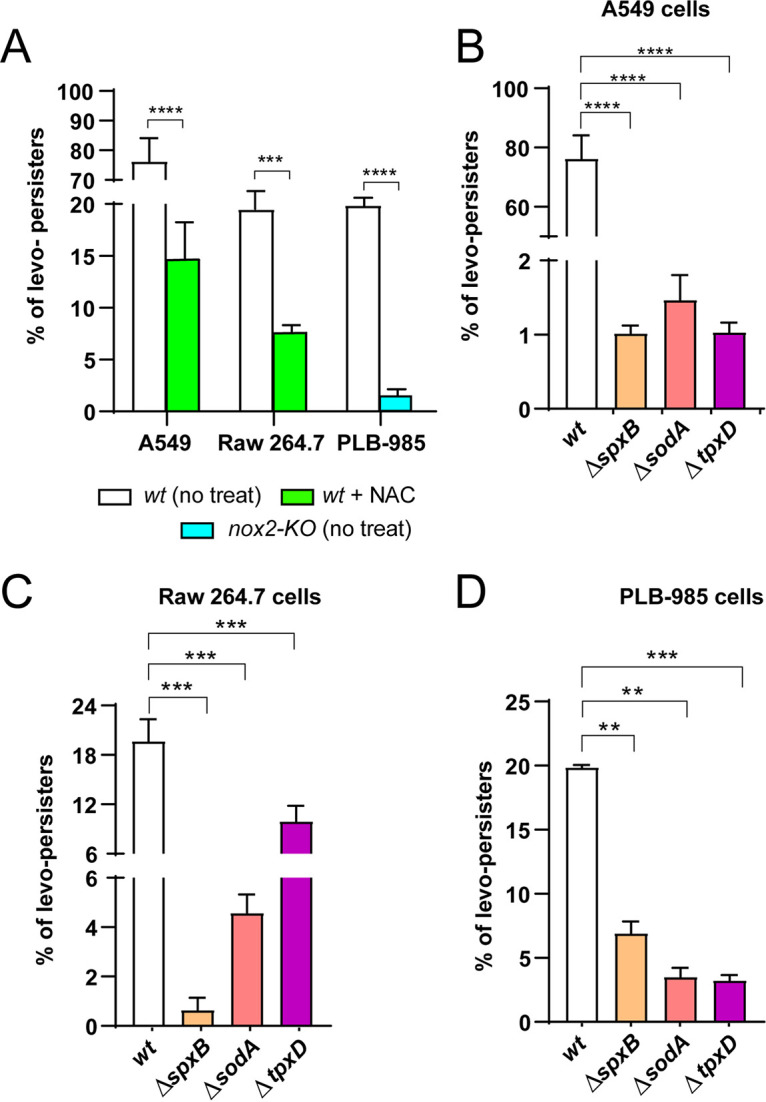
FQ persistence is induced by host cell oxidative stress and depends on the pneumococcal stress response. (A) A549 pneumocytes and Raw 264.7 macrophages were pretreated with 5 mM and 30 mM NAC, respectively, and infected with the *wt* strain using an MOI of 30:1. Non-NAC-treated cells were used as a control. Bacterial survival progression was monitored using a typical protection assay in which gentamicin was used as an extracellular antibiotic to kill nonendocytosed/nonphagocyted pneumococci. After 6 μg/mL levofloxacin treatment, samples were taken at different times according to the endocytic/phagocytic capacity of host cells. Cells were lysed by centrifugation, and CFU/mL was determined by incubation of these samples on blood agar plates at 37°C for 16 h. In addition, the differentiated PLB-985 and PLB-985-KO (*nox2* mutant with decreased ROS production) neutrophils were infected with the *wt* strain using an MOI of 30:1 (bacteria: host cells). (B, C and D) Oxidative stress genes are involved in the induction of FQ persistence in host cells. The A549 (A), Raw 264.7 (B) and PLB-985 (C) cells were infected with the Δ*spxB*, Δ*sodA*, Δ*tpxD* and *wt* strains using an MOI of 30:1 (bacteria: host cells) and exposed to 6 μg/mL levofloxacin for 4, 2 and 1 h, respectively. In all panels, the percentage of levo-persisters was calculated with the CFU values shown in Fig. S11, considering the total amount of internalized bacteria after 30 min of extracellular antibiotic treatment as representing 100%. Percentage values represent the mean ± SEM of at least three replicates. Statistical significance was determined using the two-tailed test and indicated as *P < *0.01 (**), *P < *0.001 (***) or *P < *0.0001 (****).

To determine whether ROS produced in host cells during bacterial infection can induce pneumococcal FQ persistence, we used a cellular infection model with a multiplicity of infection (MOI) of 30:1 (pneumococci: host cells), as described (15, 17). To inhibit ROS production during bacterial infection, A549 pneumocytes were treated with 5 mM NAC for 3 h (17), and Raw 264.7 macrophages with 10 mM NAC for 1 h (see infection scheme in Fig. S7). As a control, we confirmed that NAC inhibited ROS production in host cells, and it was detected by flow cytometry using H_2_DCFDA, a cell-permeant indicator for ROS (17, 42) (Fig. S8A, B and C). Subsequent to bacterial infection, cells were exposed to gentamicin, an extracellular antibiotic used to eliminate the nonendocytosed/phagocyted pneumococci (17). This point after gentamicin treatment is considered “time zero” in our infection scheme (Fig. S7). Once extracellular pneumococci were killed by gentamicin, host cells were treated with 6.0 μg/mL levofloxacin, an intracellular FQ that penetrates human soft tissues (43). Notably, we found that pneumococci-infected A549 pneumocytes and Raw 264.7 macrophages were able to generate FQ persisters (Fig. 4A and Fig. S8D, E and F). When the A549 and Raw 264.7 cells were previously treated with NAC, the ROS level decreased, as mentioned above (Fig. S8A and B), and the FQ persistence phenotype shown by the *wt* strain was significantly reduced (Fig. 4A). Additionally, we used a human promyelocytic leukemia PLB-985 cell line that can be differentiated into mature neutrophil-like granulocytes (44). We also included in this analysis *gp91^phox^* KO-PLB-985 (here referred to as PLB-985-KO), a stable cell line with *gp91^phox^* genetically knocked out that is not capable to express the gp91^phox^ subunit of the NADPH oxidase (44). More than 80% of both cell lines were differentiated into neutrophil-like granulocytes by DMSO treatment (45), which was checked by CD11b expression quantified by flow cytometry (46) (Fig. S9A) and displayed less than 2% of cell death measured by propidium iodide labeling assays (Fig. S9B). After bacterial infection, the ROS level of PLB-985-KO was reduced 3 times about PLB-985 (Fig. S8C). We evaluated FQ persistence in both cell lines, and we found that pneumococci-infected PLB-985 cells were able to generate the same percentage of FQ persisters as Raw 264.7 cells. However, this phenotype was affected in the PLB-985-KO cells, presenting a 20-fold decrease compared to PLB-985 (Fig. 4A and Fig. S8F). Overall, these results indicated that host cell oxidative stress can induce FQ persistence in S. pneumoniae.

### Pneumococcal oxidative stress genes are involved in FQ persistence induced by host cell oxidative stress.

To evaluate whether the oxidative stress genes in S. pneumoniae contribute to the FQ persistence mechanism induced by host cells, we infected A549 (Fig. S10A and B), Raw 264.7 (Fig. S10C and D), and PLB-985 cells (Fig. S10E and F) with the Δ*spxB*, Δ*sodA*, Δ*tpxD,* and *wt* strains. We observed that the percentage of levo-persisters was negatively affected in all cell lines and for all three mutants in comparison to the *wt* strain (Fig. 4A, B, and C). This finding suggests that oxidative stress genes of S. pneumoniae are also involved in the FQ-persistence mechanism induced by ROS in host cells.

## DISCUSSION

FQs have been described to promote increased production of endogenous ROS in S. pneumoniae, which are the main factors for the postantibiotic effect and contribute to the lethality of FQs (27, 29). In this study, we aimed to assess the impact of an oxidative environment on pneumococcal viability upon exposure to FQs. First, we characterized the effects of prior H_2_O_2_ exposure on pneumococcal susceptibility to different FQs such as levofloxacin, moxifloxacin, and ciprofloxacin. We observed that a high percentage of the bacterial population that survived H_2_O_2_ exposure was also competent to survive FQ treatment. As mentioned, tolerance is the ability of bacterial cells to survive transient exposure to antibiotics that would otherwise be lethal without changing the MIC (47, 48). In contrast, persistence is a type of tolerance that affects a subpopulation of bacteria that survives antibiotics (49) while the majority of the bacterial population dies (50). We found that most pneumococci died when exposed to H_2_O_2_. Remarkably, this surviving subpopulation was able to tolerate FQ concentrations similar to those in the serum of patients treated with FQ for pneumococcal infections (31, 33, 51). Based on the definitions proposed by Balaban et al. (7), we assumed that this phenotype corresponds to triggered FQ persistence in S. pneumoniae. Vancomycin tolerance (8), heteroresistance to penicillin (9) and fosfomycin (10), and resistance to FQs (52) have been described for this pathogen. Here, we report for the first time a mechanism of antibiotic persistence in S. pneumoniae.

Our first findings led us to hypothesize that the oxidative stress response of S. pneumoniae is closely related to FQ persistence. To better understand this connection, we analyzed the transcripts levels of oxidative-stress response genes and characterized the impact of the specific mutations on ROS production and FQ persistence. When bacterial cells were exposed to H_2_O_2_, we observed a raised expression of *spxB*, which encodes a pyruvate oxidase (SpxB) that is the major responsible for endogenous H_2_O_2_ production. Indeed, we observed a significant increase in intracellular H_2_O_2_ levels in pneumococci preexposed to external H_2_O_2_ reflecting the induction of the oxidative stress response. SpxB was reported to be essential for pneumococcal survival in response to H_2_O_2_ (11). Due to the pyruvate oxidase activity impacts directly carbon utilization and ATP synthesis through the glycolysis pathway, these authors hypothesized that SpxB contributes to an energy source that maintains viability during oxidative stress (19). We propose that the initial increment in the internal H_2_O_2_ production detected under oxidative stress conditions is a by-product driven by the energetic need required for pneumococcal survival.

In addition to *spxB*, many other genes have been involved in the adaptive response to endogenously produced H_2_O_2_ (41). S. pneumoniae lacks catalases to control the endogenous H_2_O_2_ levels and needs ROS-detoxifying enzymes, such as the superoxide dismutase SodA (40) and the thiol peroxidase TpxD (41), encoded by the *sodA* and *tpxD* genes, respectively, being essential for S. pneumoniae to survive in oxidative stress environments. Here, we demonstrated that these enzymes are necessary for FQ persistence development. Once these proteins are expressed, they are responsible to buffer the FQ-induced ROS production, one of the main factors by which FQ causes bacterial death.

In this work, and using fluorescent tracers in high-content analysis, we found that most subpopulations that survived H_2_O_2_ exposure had a high percentage of slow-growing and growth-arrested cells, which are considered dormant cells (7) and become persisters to FQs.

Regarding persister formation in bacteria, stress-induced persisters have been reported most frequently (53), and many stress conditions act as triggers, such as antibiotics themselves (54), as well as nutritional (55), acidic (56), osmotic (57), and oxidative (53) stresses. The antibiotic persistence triggered by oxidative stress was better described in E. coli. The OxyR transcriptional regulator, which controls the expression of antioxidant genes in response to oxidative stress in Escherichia coli, was induced by exposure to H_2_O_2_ (58) or salicylate to increase ROS production (59) and induce persisters.

To reveal the emergence of FQ persisters, we used an H_2_O_2_-treated bacterial cell assay, and this prompted us to assess the effects of host cell oxidative stress on pneumococcal persistence. Previously, we had shown that S. pneumoniae is able to survive for many hours in host cells (15–17). This survival depends on signal transduction systems that control the expression of acidic and oxidative stress genes (15, 16). In this context, the StkP/ComE pathway regulates the expression of the *spxB*, *tpxD,* and *sodA* genes and endogenous ROS production (16). Importantly, using the same pneumococcal-infected cell model (15, 16), we also detected the formation of FQ persisters in infected host cells with different ROS production levels, such as A549 pneumocytes, Raw 264.7 macrophages and PLB-985 neutrophils. We were able to inhibit FQ persistence in both A549 and Raw 264.7 cell lines with the ROS inhibitor NAC, but also in PLB-985-KO cells, which have ROS production blocked due to a mutation in *nox2* (44). Moreover, we were also able to reproduce in these cell infection models the phenotypes of Δ*spxB*, Δ*tpxD,* and Δ*sodA* mutants observed in culture media, confirming the contributions of oxidative stress genes in this persistence mechanism. These results are consistent with those observed in other bacterial genera. In Salmonella, intracellular persisters have been reported following phagocytosis by naive macrophages (56). In S. aureus, bacteria that survive antibiotic treatment in macrophages have also been reported to be persisters (60).

**FIG 5:**
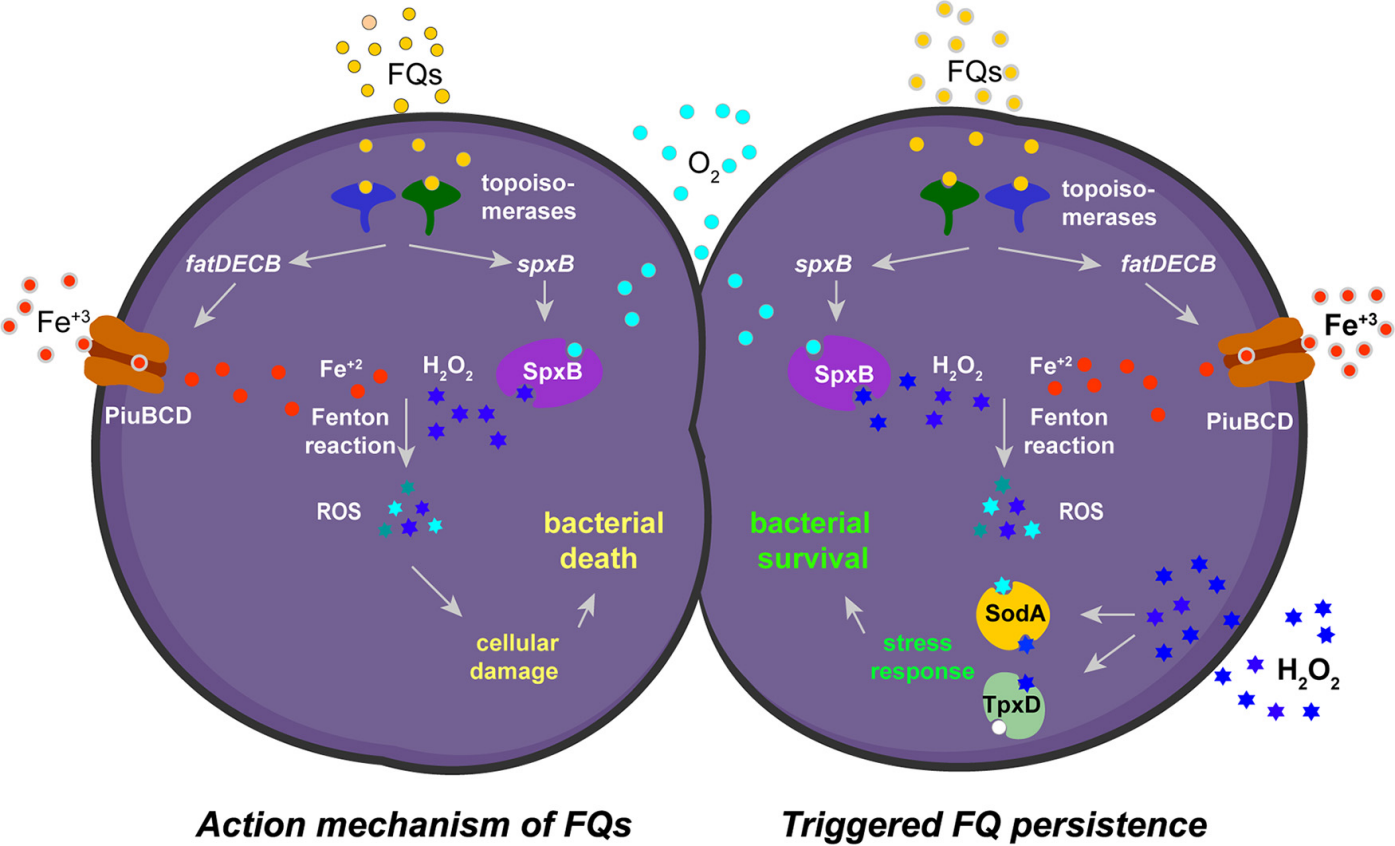
Proposed model for triggered fluoroquinolone persistence in S. pneumoniae. It was proposed that FQs inhibit topoisomerase IV, which could upregulate the expression of the *fatDCEB* operon, increasing the iron transport and the intracellular Fe^+2^ levels. The SpxB pyruvate oxidase produces endogenous H_2_O_2_, which reacts with Fe^+2^ to generate ROS via the Fenton reaction. The oxidative damage produced by ROS in proteins, DNA and lipids contributes to FQ-induced bacterial death (extracted and modified from Ferrandiz & de la Campa^29^). When pneumococci are exposed to H_2_O_2_ within host cells, the oxidative stress response of S. pneumoniae induces the expression of ROS detoxifying enzymes, such as the SodA superoxide dismutase and the TpxD thiol peroxidase, to protect them from FQ-induced oxidative damage. This adaptive response allows the pneumococcus to generate FQ persisters and to survive.

One possible explanation for the development of FQ persistence is that a previous oxidative shoot produces an SpxB-mediated rise in endogenous H_2_O_2_ that triggers the pneumococcal oxidative stress response. In contrast to other antibiotic persistence induced by oxidative stress through indole (58, 59) and cyclic AMP signaling (61) in E. coli, or negatively affected by catalase and SodA activity in *P. aeruginosa* (55), this adaptive mechanism is mainly supported by ROS detoxifying enzymes such as SodA and TpxD, which protect S. pneumoniae from the high ROS levels induced by FQ and favor the generation of FQ persisters and the subsequent survival of S. pneumoniae to these antibiotics (Fig. 5).

In addition to this active stress response, we propose that FQ persistence is also mediated, in a complementary and synergistic manner, by the growth arrest effects caused by H_2_O_2_. In this sense, it has been described that mutant bacteria deficient in the starvation-signaling stringent response increase the production of antioxidant enzymes (SodA and catalase) to form drug persisters in Pseudomonas aeruginosa (55).

It is well known that bacterial stress responses lead to the induction of error-prone polymerase and the emergence of adaptive mutations (62). Of concern, stress-induced persisters have been shown to undergo DNA damage (63), and prolonged exposure to antibiotics likely favors the acquisition of mutations that generate antibiotic resistance in persisters (53). Indeed, Escherichia coli populations derived from FQ persisters had greater amounts of antibiotic-resistant mutants than those from untreated controls, suggesting that FQ persisters accelerate the development of antibiotic resistance (30). Given these observations, assays to determine the emergence of FQ resistance among persisters in S. pneumoniae are currently being developed in our laboratory.

As mentioned, FQs are considered an important treatment option for invasive pneumococcal disease (64); however, the emergence of FQ-resistant strains complicates antibiotic treatment of invasive infections (6). Here, we propose that persistence induced by host cells, particularly macrophages and neutrophils, would be part of a multistep FQ resistance evolution, as described in other pathogens, but this possibility remains to be demonstrated.

## MATERIALS AND METHODS

### Bacterial and growth conditions.

All bacterial strains used in this study, as well as oligonucleotides used for mutagenesis procedures, are listed in the supplemental material (Table S1). Oligonucleotide synthesis and DNA sequencing services were performed at Macrogen Inc. (Seoul, South Korea). The growth conditions and stock preparation of the pneumococcal strains, as well as the DNA transformation procedures, have been previously reported (65, 66).

### Antibiotic-survival assay.

The pneumococcal strains were grown in BHI to midlog phase, first exposed to 20 mM H_2_O_2_ and then to either 6 μg/mL levofloxacin, 5 μg/mL moxifloxacin, 2.5 μg/mL ciprofloxacin, or 10 μg/mL rifampicin for 5 h. Here, each time that pneumococcal strains cells are exposed to H_2_O_2_ or NAC, then bacterial cells are centrifuged, washed with PBS, and resuspended in culture media. Aliquots were removed at different time points, and dilutions were seeded on blood agar plates. Survivors were considered FQ-persisters and expressed as CFU/mL. MICs were determined by the broth microdilution method recommended by the Clinical and Laboratory Standards Institute (CLSI) (67).

### Hydrogen peroxide determination in S. pneumoniae.

Cells were grown in C+Y medium (68) to the midlog phase and exposed to either 20 mM H_2_O_2_ for 30 min, 10 mM NAC for 1 h, or 10 mM NAC for 1 h followed by 20 mM H_2_O_2_ for 30 min. Then, bacterial cultures were exposed to 6 μg/mL levofloxacin for 5 h. Aliquots were taken and serially diluted to determine viable cells by plating on blood agar. In parallel, other aliquots were transferred to multiwell plates and mixed with the same volume of PRS buffer (NaCl 140 mM, dextrose 5.5 mM, 280 μM phenol red, and 8.5 U/mL horseradish peroxidase in PBS pH 7.0). Reaction mixtures were incubated at 37°C for 90 min and stopped with 10 μL of 1 N NaOH. The reactive wells were read in a microplate reader (Bio-Rad) using the 595 nm filter. Assays were performed in triplicate and results are expressed as millimoles of H_2_O_2_ released by 1× 10^6^ cells (16). The doubling time was determined as described previously (69).

### Cell lines and culture conditions.

The A549 cell line (human lung epithelial carcinoma, pneumocytes type II; ATCC CCL-185) and Raw 264.7 (ATCC TIB-61) were cultured at 37°C, 5% CO_2_ in DMEM with 4.5 g/l of glucose, 1% penicillin/streptomycin (P/S), and 10% of heat-inactivated fetal bovine serum (FBS) (Gibco BRL, Gaithersburg, MD). The undifferentiated human monomyelocyte PLB-985 and *gp91^phox^* KO-PLB-985 (PLB-985-KO) cells (44) were cultured in a complete medium RPMI 1640, supplemented with 1% P/S, and 10% heat-inactivated FBS. All cell lines were regularly tested for *Mycoplasma*, *Acholeplasma*, and *Ureaplasma* contamination as described (70).

### Quantification of ROS levels in host cells.

ROS were detected using 10 μM H_2_DCFDA (2′,7′ dichlorodihydrofluorescein diacetate Sigma D6883), a peroxide-sensitive fluorescent probe (42), by a cytometry flow analysis (Beckton Dickinson FACSCanto II). To discount dead cells, they were incubated with 5 μg/mL propidium iodide. Results were analyzed in Flow Jo V 7.6.2 software, and only living cells were considered.

### Neutrophil differentiation by DMSO treatment.

The differentiation of PLB-985 and PLB-985-KO cells into the neutrophil-like phenotype was performed as reported (45). The cell surface expression of CD11b was quantified to confirm the differentiation of PLB-985 and PLB-985-KO to neutrophils, as described (43, 46), using antihuman CD11b monoclonal antibody (ICRF44; eBioscience) and antimouse/Alexa Fluor 647 (BioLegend). The fluorescence was analyzed by flow cytometry (Attune NxT, ThermoFisher Scientific).

### Determination of FQ persistence of S. pneumoniae in host cells.

The intracellular survival assays of pneumococci were performed as reported previously (15, 16). For the treatment with NAC (Sigma A9165), A549 cells were incubated with 5 mM NAC for 3 h, while Raw 264.7 cells were incubated with 10 mM NAC for 1 h. The NAC treatment was carried out in parallel to the bacterial infection protocol, as described (17). Apoptosis/necrosis was quantified by flow cytometry using a propidium iodide labeling kit (ThermoFisher). The cell infection scheme was carried out as described in Fig. S7.

To analyze the emergence of FQ-persistent pneumococci, the bacterial-infected A549 and Raw 264.7 cells were cultured in DMEM-1% FBS-6μg/mL levofloxacin. The pneumococci-infected PLB-985 and PLB-985-KO cells were cultured in RPMI 1640/1% SFB/1,3% DMSO/6 μg/mL levofloxacin. Cell cultures were incubated at 37°C and 5% CO_2_ at different time points. Cells were lysed by centrifugation for 10 min at 15,000 g and the bacterial pellet was resuspended in BHI. The number of internalized bacteria was quantified after serial dilutions of lysates and plating on blood agar plates. The number of surviving bacteria obtained at t0 was defined as 100% survival in all the cases, and the data obtained at different time points were used to calculate the respective percentages of FQ persisters.

### Quantitative PCR.

Bacterial cells were grown in BHI to the midlog phase and exposed to 20 mM H_2_O_2_ for 30 min. Then, RNA purification, cDNA synthesis and qPCR were performed as described (16). Genes were amplified using the oligonucleotides listed in Table S1 and PowerUp SYBR green Master Mix following the manufacturer’s protocol (Applied Biosystem). The *gyrB* was used to normalize the expression in S. pneumoniae using the ΔΔCt method (71), as described previously (16).

### Growth curves analysis of S. pneumoniae.

Aliquots of overnight cultures were subcultured in BHI, grown to midlog phase and exposed to either 20 mM H_2_O_2_, 2 μg/mL chloramphenicol, or acidified medium (pH 5.2). The bacterial cells were centrifuged and resuspended in BHI. Aliquots of 100 μL were plated in 96 wells and incubated at 37°C for 16 h, and OD_600nm_ was measured every 15 min (BioTek H1 Microplate Reader). Assays were performed in triplicate.

### Analysis of the arrested growth of S. pneumoniae using high-content microscopy.

Pneumococci were exposed to carboxyfluorescein diacetate succinimidyl ester (CFDA-SE; Sigma 21878). After H_2_O_2_ or FQ treatment, pneumococci were exposed to 50 μg/mL propidium iodide and 1.0 μg/mL Hoechst for 5 min, and samples were mounted on glass slides. The bacterial images were acquired *in vivo* using either an epifluorescence microscope (Leica DMI 8) or a high-content screening (InCell Analyzer 2000, GE Healthcare). The percentage of green bacteria versus red bacteria was calculated using the In-Cell Investigator software V1.5 (GE Healthcare).
