# Supplementary material for: Host Cell Oxidative Stress Promotes Intracellular Fluoroquinolone Persisters of Streptococcus pneumoniae
Source: Microbiol Spectr. 2022 Nov 29;10(6):e04364-22. doi: 10.1128/spectrum.04364-22 (PMC9769771; doi:10.1128/spectrum.04364-22)
Supplement: Supplemental file 1 — Fig. S1 to S10 and Table S1. Download spectrum.04364-22-s0001.pdf, PDF file, 1.3 MB [file spectrum.04364-22-s0001.pdf]

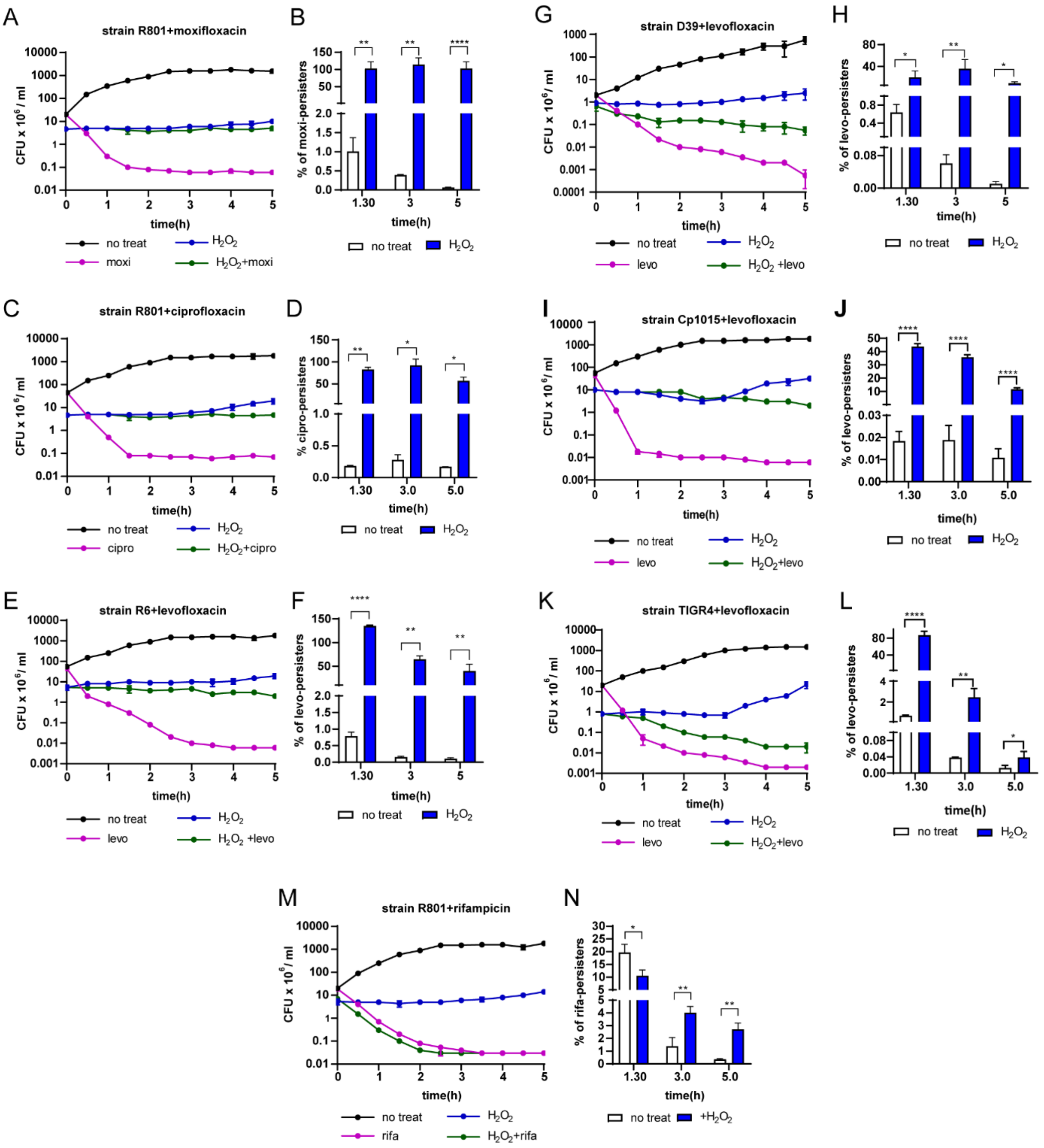

FIG S1 Oxidative stress promotes persistence to different fluoroquinolones and in different pneumococcal strains. The R801 (a variant of R6), R6 (a non-capsulated variant of D39), D39 Δ*cpsB* (a non-capsulated variant of D39, serotype 2), Cp1015 (a non-capsulated variant of Rx, serotype 3) and TIGR4 (capsulated, serotype 4) strains were grown in BHI to mid-log phase and exposed to 20 mM H_2O_2 for 30 minutes before adding either 5 µg/ml moxifloxacin (A and B), 2.5 µg/ml ciprofloxacin (C and D), 6 µg/ml levofloxacin (E-L) or 10 µg/ml rifampicin (M and N) for 5h. After 5 h of antibiotic treatment, samples were removed at the indicated time points. Bacterial cells were collected by centrifugation, washed in PBS, resuspended, and seeded into blood agar plates to determine bacterial survival. Growth curves represent the CFU/ml values found at different time points of the bacterial cultures. In the B, D, F, H, J, L and N panels, the percentage of antibiotic persisters in H_2O_2-treated or non-treated cells is

shown. Percentage values were obtained from data shown in panels A, C, E, G, I, K and M and represent the mean ± SEM of at least three replicates. Statistically significant differences were determined using the two-tailed test and are indicated as $p < 0.05$ (*), $p < 0.01$ (**) or $p < 0.0001$ (****).

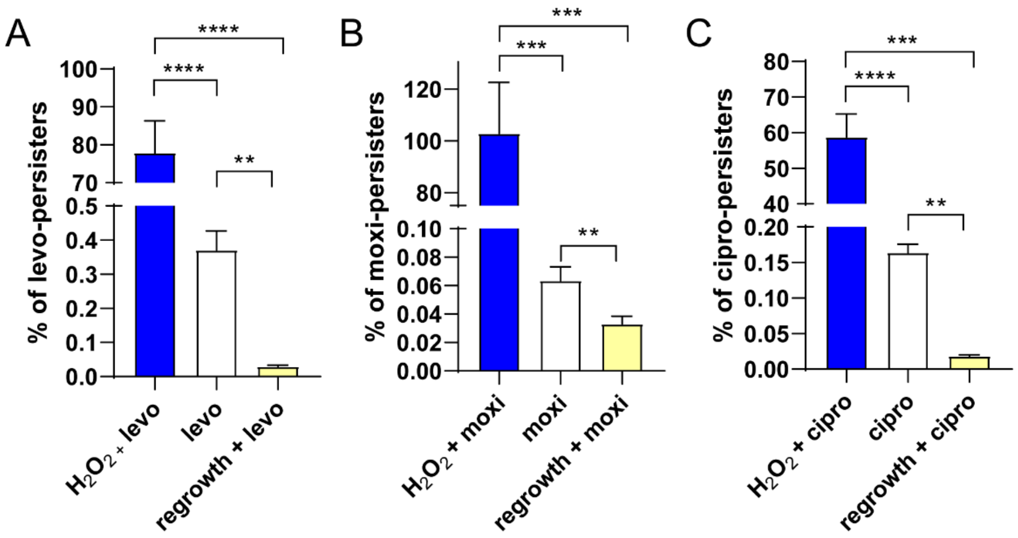

FIG S2 Susceptibility to fluoroquinolones is restored in persisters after regrowth. To confirm that FQ persistence is a transient phenotype, the R801 strain was grown in BHI to mid-log phase and exposed to 20 mM H_2O_2 for 30 minutes before adding either 6 µg/ml levofloxacin (A), 5 µg/ml moxifloxacin (B), or 2.5 µg/ml ciprofloxacin (C) for 5 h. After 5 h of antibiotic treatment, samples were removed at the indicated time points. Bacterial cells were collected by centrifugation, washed in PBS, resuspended, and seeded into blood agar plates to determine bacterial survival. Aliquots of each culture were regrown in BHI to mid-log phase and exposed again with the antibiotic used initially for 5 h, and bacterial cells were treated as mentioned to determine CFU. The percentage of antibiotic persisters in H_2O_2-treated or non-treated cells is shown and represents the mean ± SEM of at least three replicates. Statistically significant differences were determined using the two-tailed test and are indicated as $p < 0.05$ (*), $p < 0.01$ (**) or $p < 0.0001$ (****).

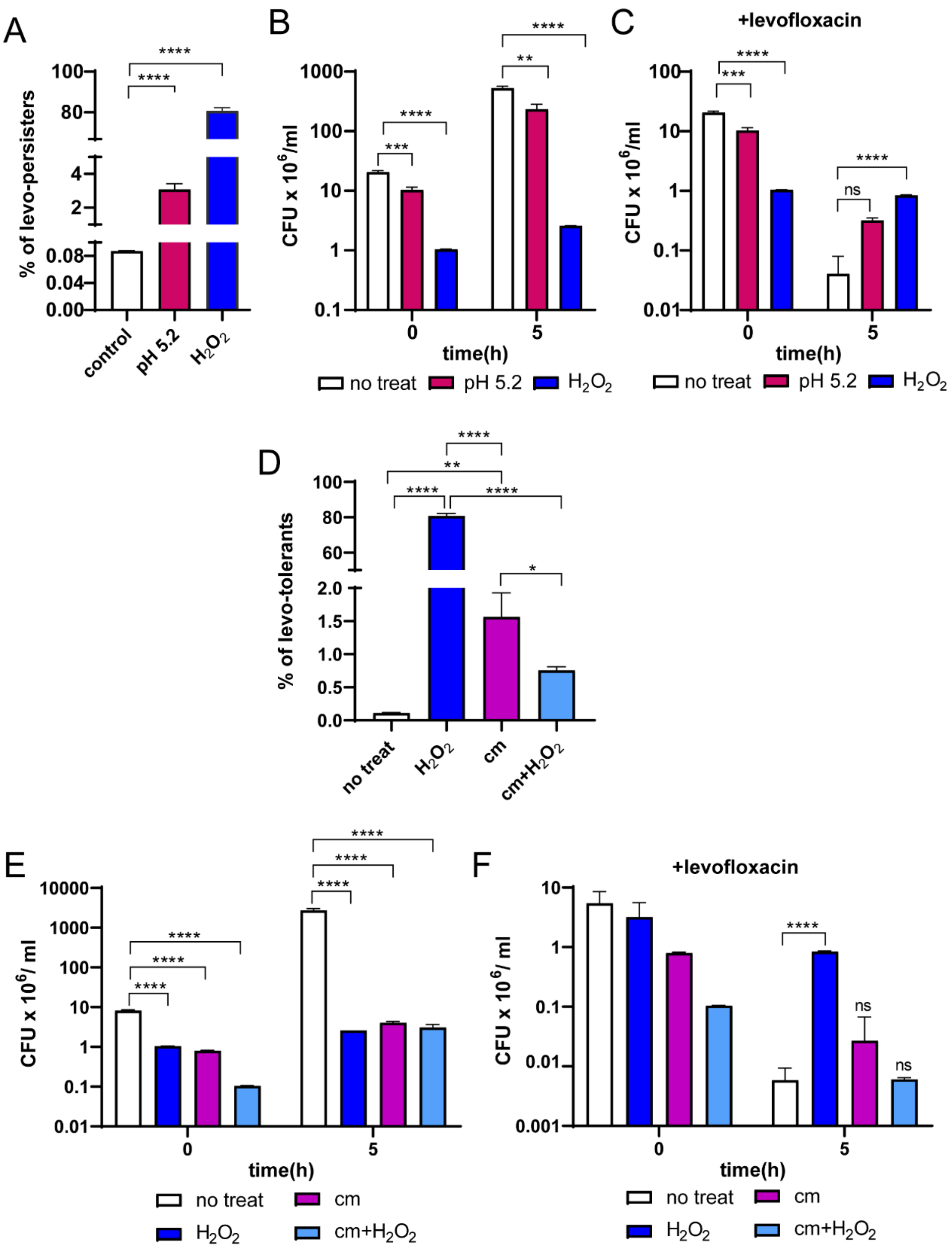

FIG S3 FQ persistence under different stress conditions. (A) FQ persistence is poorly induced by acidic stress conditions. The *wt* strain was grown in BHI to mid-log phase before being exposed to either 20 mM H_2O_2 for 30 minutes or MD medium (pH 5.2) for 2h. Bacterial cells were collected by centrifugation, washed with PBS, resuspended in BHI and exposed to 6 µg/ml levofloxacin for 5 h. Non-treated cells were used as control. The number of viable cells was measured as described in Fig S1. Percentages were calculated with the CFU/ml values shown in the b and c panels. (B) CFU/ml values correspond to cultures without antibiotic treatment. (C) CFU/ml values corresponding to levofloxacin-treated cultures after 5 h of exposure. (D) FQ persistence mechanism is dependent on protein synthesis. The *wt* strain was grown

in BHI to mid-log phase and exposed to either 2 µg/ml chloramphenicol (a typical protein-synthesis inhibitor) for 1 h, 20 mM H_2O_2 for 30 minutes, or 2 µg/ml chloramphenicol for 1 h followed by the addition of 20 mM H_2O_2 for 30 minutes. Posteriorly, bacterial cells were collected by centrifugation, washed with PBS, resuspended in BHI and exposed to 6 µg/ml levofloxacin for 5 h. Then, the number of viable cells was measured as described in Fig S1. The percentage of levo-persisters in cultures treated with different stressors was calculated with the CFU/ml values shown below. (E) CFU/ml values were obtained at different time points in cultures after stressor exposure but without levofloxacin treatment. (F) CFU/ml values were obtained at different time points in cultures following stressor exposure and treatment with 6 µg/ml levofloxacin for 5 h. For all panels, values represent the mean ± SEM of at least three replicates. Statistically significant differences were determined using the two-tailed test and are indicated as $p < 0.01$ (**), $p < 0.001$ (***) or $p < 0.0001$ (****).

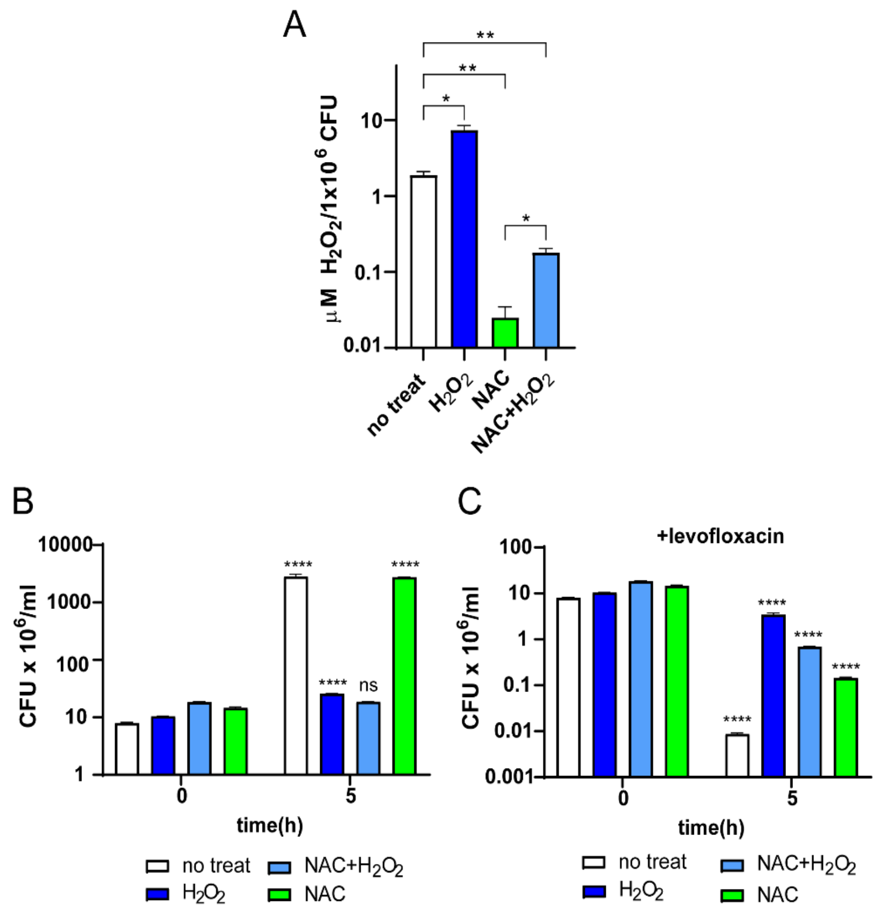

FIG S4 NAC treatment inhibits H_2O_2 production in *S. pneumoniae*. (A) The *wt* strain was grown in BHI until the mid-log phase and exposed to either 20 mM H_2O_2 for 30 minutes, 10 mM NAC (inhibitor of ROS production) for 1 h, or 10 mM NAC for 1 h followed by the addition of 20 mM H_2O_2 for 30 minutes. The H_2O_2 concentration was measured by the horseradish peroxidase method. Values are expressed in µM and normalized against $1x10^6$ viable cells. (B) The bacterial cells were cultured as mentioned in panel a. The number of viable cells was measured as described in Fig S1. (C) The bacterial cells were cultured as mentioned in panel a. Posteriorly, each culture was exposed to 6 µg/ml levofloxacin for 5h. The number of viable cells was measured as described in Fig S1. The data shown in the a-b panels correspond to the determination of the percentage of levo-persisters shown in Fig 1. Values represent the mean ± SEM of at least three replicates. Statistically significant differences were determined using the two-tailed test and are indicated as $p < 0.05$ (*), $p < 0.01$ (**) or $p < 0.0001$ (****).

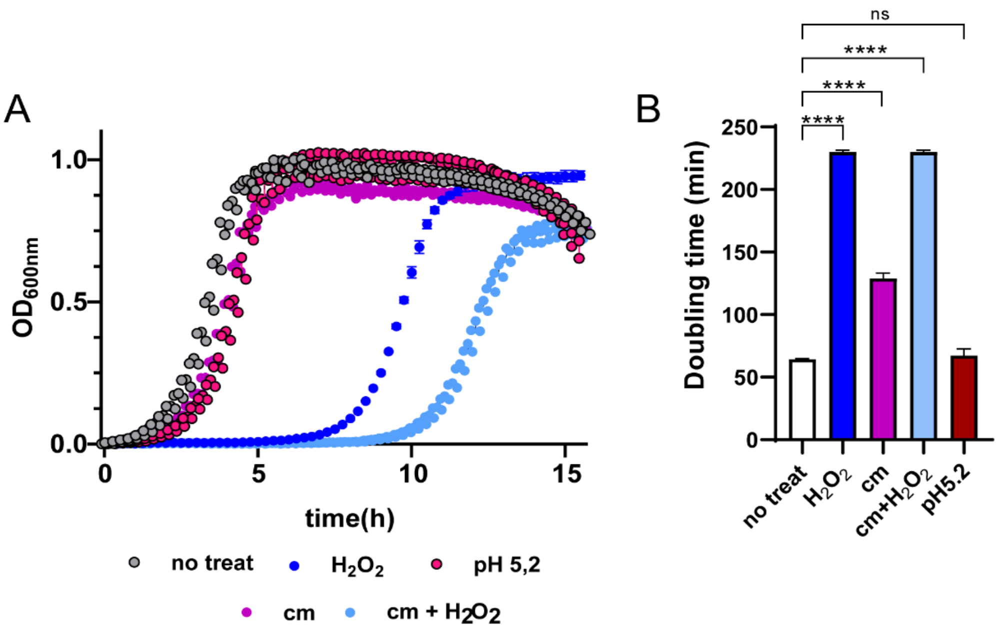

FIG S5 H_2O_2 treatment increases the doubling time of the pneumococcal cells. (A) The *wt* strain was grown in BHI to mid-log phase and then exposed to either 20 mM H_2O_2, or 2 µg/ml chloramphenicol, or 2 µg/ml chloramphenicol for 1 h followed by the addition of 20 mM H_2O_2. In parallel, bacterial cells were collected by centrifugation and resuspended in MD at pH 5.2. All cultures were diluted 1/10 in the same bacterial media, and optical density at 600 nm was measured at different time points to draw the corresponding growth curves. (B) The doubling time of the *wt* strain cultured under different stress conditions was determined using the data shown in panel a, and it was calculated using the number of CFU/ml obtained at 0 and 5 h of exposure. Values represent the mean ± SEM of at least three replicates. Statistically significant differences were determined using the two-tailed test and are indicated as $p < 0.0001$ (****).

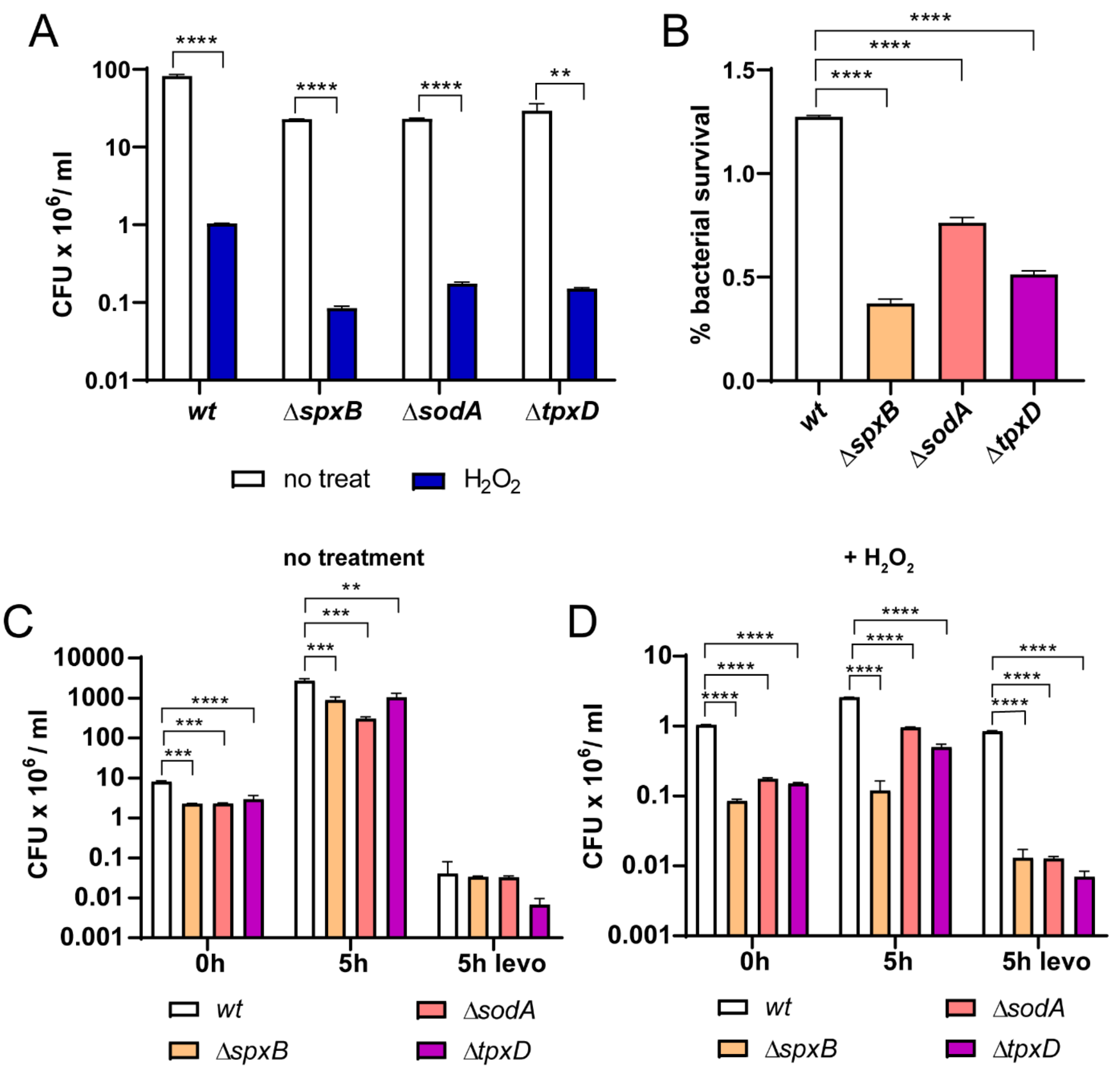

FIG S6 The Δ*spxB,* Δ*sodA,* and Δ*tpxD* mutants are more susceptible to H_2O_2 and generate fewer FQ persisters than the *wt* strain. (A) The Δ*spxB,* Δ*sodA,* Δ*tpxD* and *wt* strains were grown in BHI to mid-log phase and exposed to 20 mM H_2O_2 for 30 minutes. The number of viable cells was measured as described in Fig S1. (B) The percentage of bacterial survival was calculated with the data shown in panel A. To determine FQ persistence, the Δ*spxB,* Δ*sodA,* Δ*tpxD* and *wt* strains were grown in BHI to mid-log phase without any treatment (C) or exposed to 20 mM H_2O_2 for 30 minutes (D). Bacterial cells were collected by centrifugation, resuspended in BHI and exposed to 6 µg/ml levofloxacin for 5 h. The number of viable cells was measured as described in Fig S1. These CFU/ml values were used to determine the percentage of levo-persisters shown in Fig 3. Values represent the mean ± SEM of at least three replicates. Statistically significant differences were determined using the two-tailed test and are indicated as $p < 0.01$ (**), $p < 0.001$ (***) or $p < 0.0001$ (****).

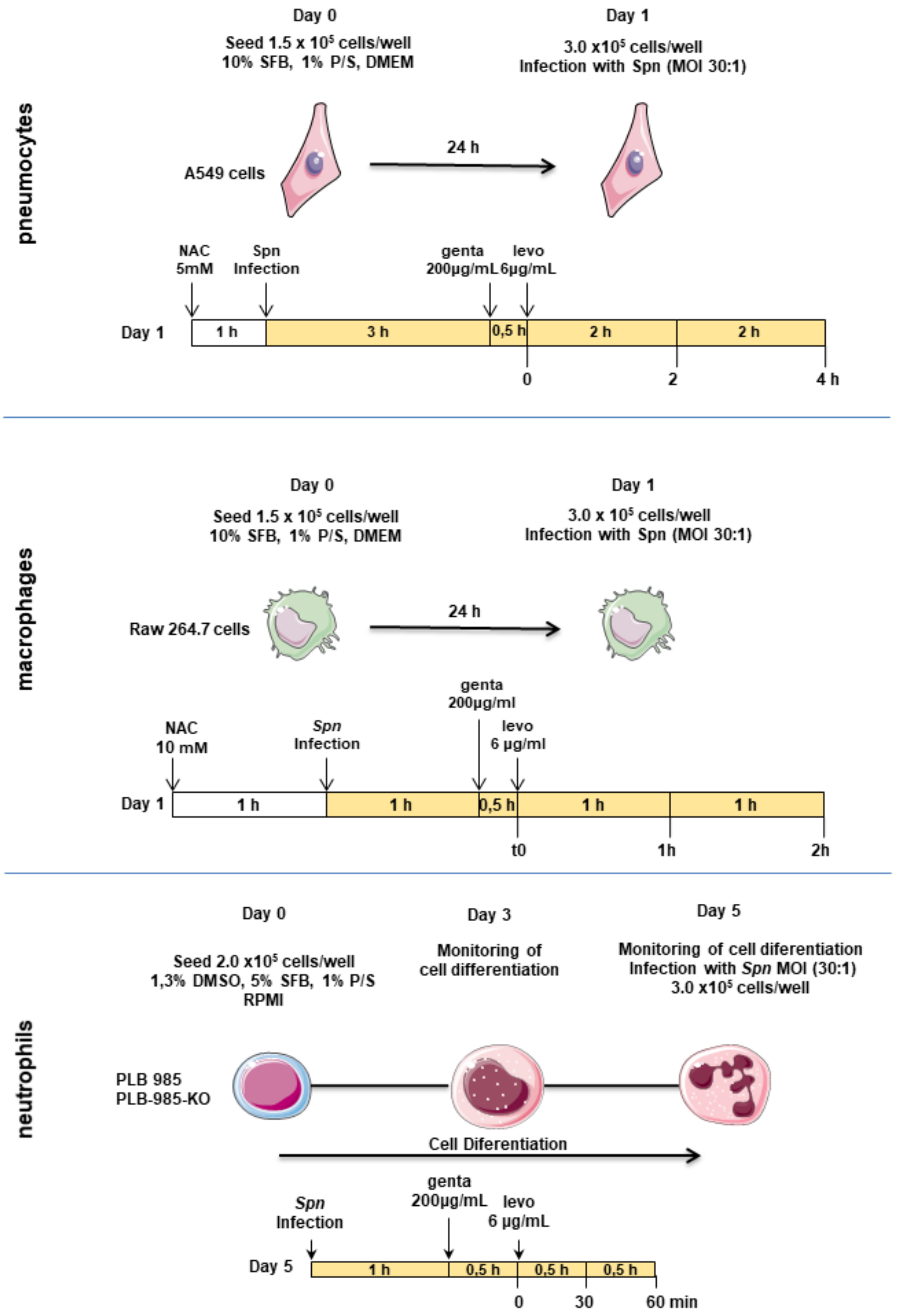

FIG S7 Host cell models for pneumococcal infection. A549 pneumocytes (A), Raw 264.7 macrophages (B) and differentiated PLB-985/PLB-985-KO neutrophils (C) were cultured into multi-well plates with DMEM/ 10% FBS/ 1% penicillin/streptomycin and incubated at 37ºC and 5% CO_2. After 16 h, the culture medium was replaced and cells were infected with the pneumococcal strain at an MOI 30:1 (bacterial: host cells). When it was indicated, NAC (5 mM for A549 cells; 10 mM for Raw 264.7 cells) was added 1 h before the bacterial infection. After gentamycin treatment to kill extracellular pneumococci, cells were lysed by centrifugation. Samples were diluted with BHI, seeded into blood agar plates and incubated at 37ºC for 16 h to count CFU/ml. This figure was partly generated using Servier Medical Art, provided by Servier, licensed under a Creative Commons Attribution 3.0 unported license.

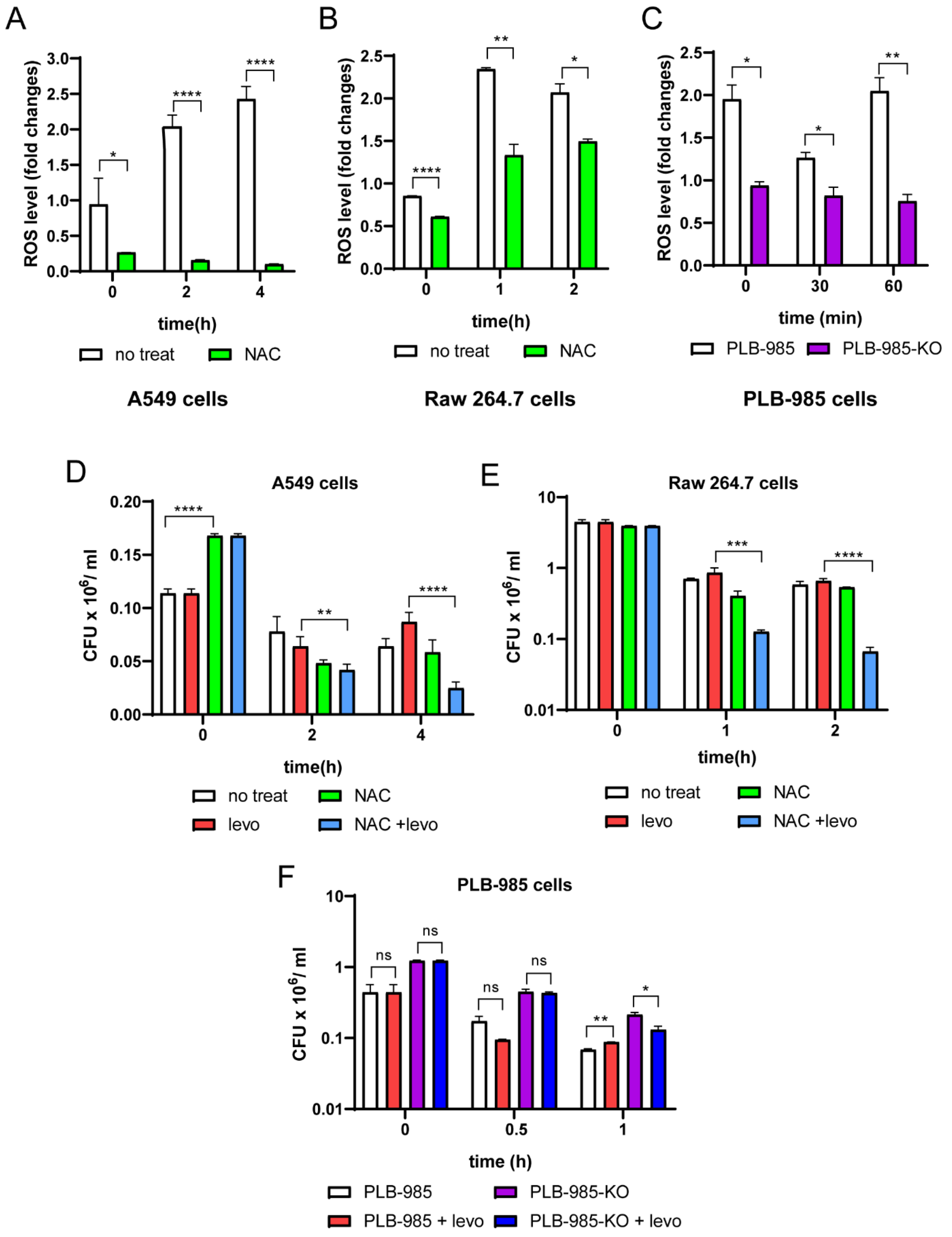

FIG S8 FQ persistence depends on the intracellular ROS levels in host cells. To determine ROS levels in host cells, A549 pneumocytes (A), Raw 264.7 macrophages (B), and differentiates PBL-985/PLB-985-KO neutrophils (C) were cultured into multi-well plates with DMEM/ 10% FBS/1% penicillin/ streptomycin and incubated at 37ºC and 5% CO_2. After 16 h, the culture medium was replaced and cells were infected with

the *wt* strain at an MOI of 30:1 (bacterial: host cells). At different time points, samples were removed and treated with 10 µM H_2DCFDA and 50 µg/ml propidium iodide for 30 minutes. Then, cells were analysed by flow cytometry to quantify ROS levels. The fold changes of ROS levels were expressed as fluorescence intensity of DCF(+)/IP(-)-cells infected at indicated times. To determine FQ persistence in host cells, the A549 pneumocytes (D) and Raw 264.7 macrophages (E) were pre-treated with either 5 mM or 10 mM NAC, respectively, and infected with the *wt* strain using an MOI of 30:1 (bacteria: host cells). Non-NAC-treated cells were used as control. The differentiated PLB-985 and PLB-985-KO (*nox2* mutant with decreased ROS production) neutrophils (F) were infected with the *wt* strain using an MOI of 30:1 (bacteria: host cells). Bacterial survival progression was monitored using a typical protection assay, as described in Fig S7, in which gentamicin was used as an extracellular antibiotic to kill non-endocytosed/non-phagocyted pneumococci. After 6 µg/ml levofloxacin treatment, samples were taken at different times according to the endocytic/phagocytic capacity of host cells. Cells were lysed by centrifugation, and CFU was determined by incubation of these samples on blood agar plates at 37˚C for 16 h. The CFU values were used to calculate the FQ persistence percentage shown in Fig 4. Values represent the mean ± SEM of at least three replicates. Statistically significant differences were determined using the two-tailed test and are indicated as $p < 0.05$ (*), $p < 0.01$ (**), $p < 0.001$ (***) or $p < 0.0001$ (****).

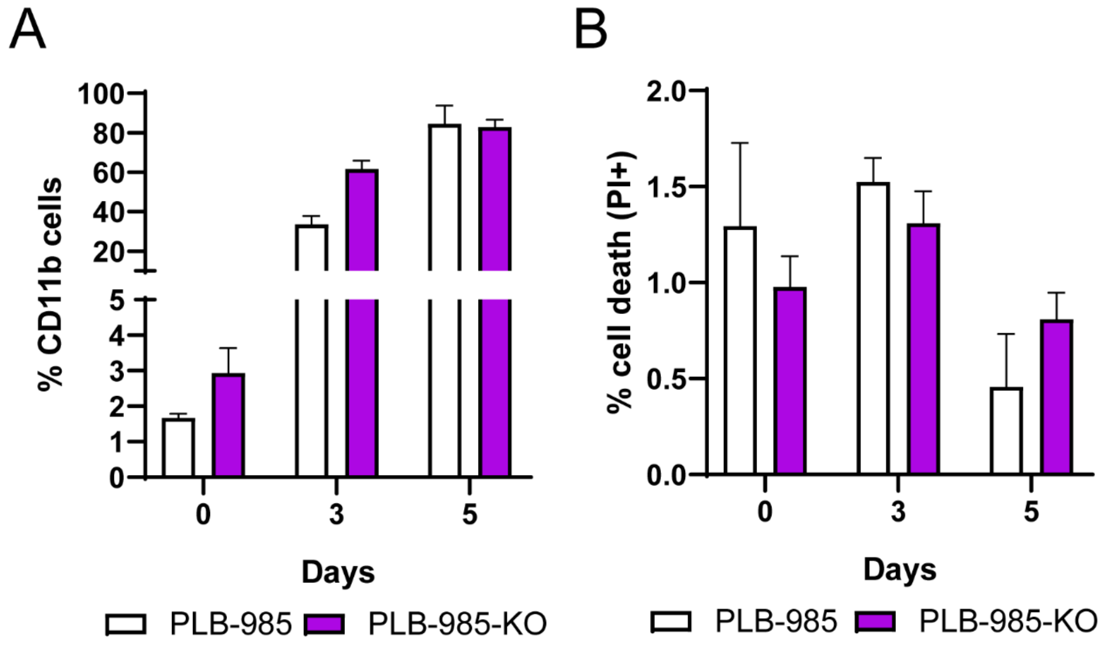

FIG S9 The PBL-985 and PLB-985-KO cells were efficiently differentiated after DMSO treatment. The PLB-985 and PLB-985-KO cells were cultured with RPMI culture medium supplemented with 5% FBS, 1% penicillin/streptomycin, and 1.3% DMSO. Cells were differentiated into neutrophils after 5 days. As a control, undifferentiated PLB-985 and PLB-985-KO cells were cultured in the absence of DMSO. To quantify the cell differentiation and cell death percentages, cells were stained with anti-CD11b (A**)** and propidium iodide (B) and analysed by flow cytometry during the process on days 0, 3 and 5. Values represent the mean ± SEM of at least three replicates. Statistically significant differences were determined using the two-tailed test and are indicated as $p < 0.05$ (*), $p < 0.01$ (**), $p < 0.001$ (***) or $p < 0.0001$ (****).

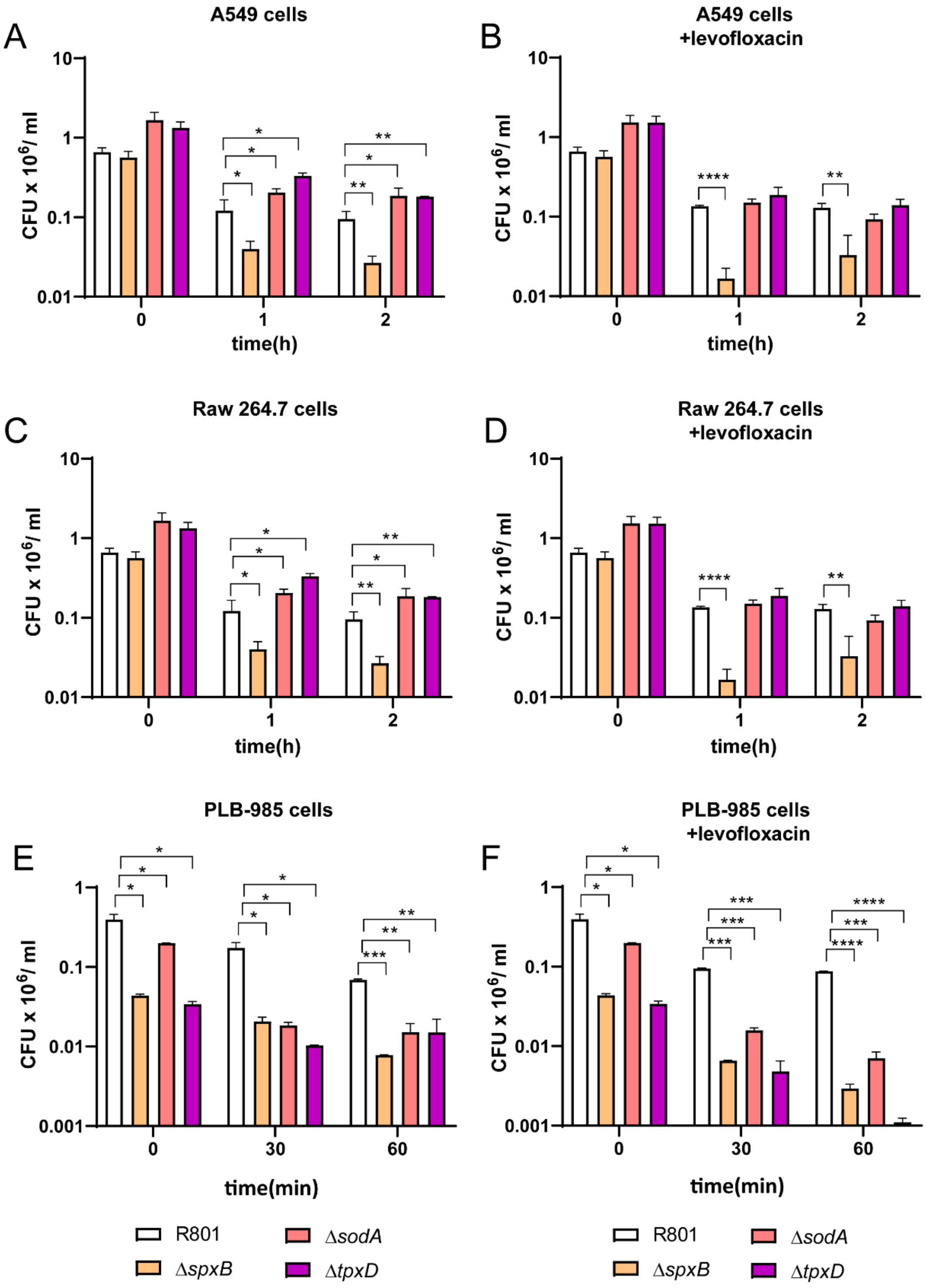

FIG S10 The *spxB*, *sodA*, and *tpxD* genes are involved in the induction of FQ persistence in host cells. The A549 (A and B), Raw 264.7 (C and D) and PLB-985 (E and F) cells were infected with the Δ*spxB*, Δ*sodA*, Δ*tpxD* and *wt* strains using an MOI of 30:1 (bacteria: host cells). The number of levo-persisters was determined as described in the Fig 4 legend. As a control, host cells were not treated with levofloxacin. The CFU values obtained in all panels were used to calculate the percentage of FQ persisters shown in Fig 4. Values represent the mean ± SEM of at least three replicates. Statistical significance was determined using the two-tailed test and indicated as $p < 0.05$ (*), $p < 0.01$ (**), or $p < 0.0001$ (****).

Table S1 Bacterial strains and primers used in this work

Name	***Relevant characteristics***	***References***
**Strains**		
Cp1015	Derivate of Rx1 (serotype 3)	1
R6	Derivate of D39	ATCC 6303
R801	Derivative of R6; *hexB* Sm^S	2
R806	R801, but *rpsL1* obtained by transformation of PCR product amplified by FrpsL and RrpsL primers from the CP1296 chromosomal DNA; Sm^R	3
D39 *cpsB::ery*	D39 but *cpsB::ery,* Ery^R (serotype 2)	3
Δ*sodA*	R806, but Δ*visR:: kan-rpsL⁺,* obtained by Janus cassette system (amplified from CP1296 chromosomal DNA) and pneumococcal DNA amplified with the FsodA1, RsodA1, FsodA2 and RsodA2 primers. Km^R, Sm^S.	3
Δ*tpxD*	R806, but Δ*tpxD:: kan-rpsL⁺,* obtained by Janus cassette system (amplified from CP1296 chromosomal DNA) and pneumococcal DNA amplified with the FtpxD1, RtpxD1, FtpxD2 and RtpxD2 primers. Km^R, Sm^S.	This work
Δ*spxB*	R806, but Δ*spxB:: kan-rpsL⁺,* obtained by Janus cassette system (amplified from CP1296 chromosomal DNA) and pneumococcal DNA amplified with the FspxB1, RspxB1, FspxB2 and RspxB2 primers. Km^R, Sm^S.	This work
TIGR4	Serotype 4	ATCC BAA-334

Primers	***DNA sequences (5'-3')***	***Amplified gene***
FJanus2	TTGGATCCGCTAGCCTCGAGAAGCTTGGAACAAGTTATTACTTGAAGATGTCAG	*Janus cassette*
RJanus2	AAGTCGACATCGATAGATCTTCTAGACCCTTTCCTTATGCTTTTGGAC	*Janus cassette*
FtpxD1-J	TGCAATCATTCTTGTAGCATGTGC	*tpxD*
RtpxD1-J	AATCAAAGATCTAAAGCTTGAGAAAAGTTACCATAGGATACTCCAATCT	*tpxD*
FtpxD2-J	ATATCAGGATCCAAGCTTAAAATCCTCTGTCACAAAGAGTTCCC	*tpxD*
RtpxD2-J	ATGACAATCTTTGGTGTGGCAGGT	*tpxD*
F1M-spx	TGTTTCAGGGCGAATCAATG	*spxB*
R1M-spx	GTTGTTGTCGACGGATCCTTATTACAGATGCAGTAATTTTCCCTTGAGT	*spxB*
F2M-spx	GTTGTTGTCGACGGATCCCGTACCATTCCGTCTCTTCTTG	*spxB*
R2M-spx	GCTGTTGATGCCCATACAGG	*spxB*
FgyrB-RT	AGCCCACATTCGTACCCTTCT	*gyrB*
RgyrB-RT	CGCTTCCAACCTTGACACCATAG	*gyrB*
FspxB-RT	GCTCATTCATCGCTCCTGC	*spxB*
RspxB-RT	GATAACTGGGCGTTCAGCATTG	*spxB*
FsodA-RT	AGCCTTGCTTGCTGATGTAGAATC	*sodA*
RsodA-RT	AAGTGTCCGCCACCATTGTTG	*sodA*
FtpxD-RT	TGGCTGGACTGGACAACACG	*tpxD*
RtpxD-RT	CGCCCGAAAGAATGGTCAAAGTA	*tpxD*

Abbreviations: EryR: erithromycin resistance; KmR: kanamycin resistance; StrR: streptomycin resistance.

References

1) Morrison, D. A., Lacks, S. A., Guild, W. R. and Hageman, J. M. (1983). Isolation and characterization of three new classes of transformation-deficient mutants of Streptococcus pneumoniae that are defective in DNA transport and genetic recombination. J. Bacteriol.156, 281-290.

2) Lefevre JC, Claverys JP, Sicard AM. Donor deoxyribonucleic acid length and marker effect in pneumococcal transformation. 1979. *J. Bacteriol*. 138:80-6.

3) Reinoso-Vizcaino NM, et al. The pneumococcal two-component system SirRH is linked to enhanced intracellular survival of Streptococcus pneumoniae in influenza-infected pulmonary cells. *PLoS Pathog* 16, e1008761 (2020).
